# Effect of long-term application of pig slurry and NPK fertilizers on trace metal content in the soil

**DOI:** 10.1007/s11356-024-34993-1

**Published:** 2024-10-04

**Authors:** Przemysław Barłóg, Lukáš Hlisnikovský, Remigiusz Łukowiak, Eva Kunzová

**Affiliations:** 1 Department of Agricultural Chemistry and Environmental Biogeochemistry, Poznań University of Life Sciences, Wojska Polskiego 71F, 60-625 Poznań, Poland; 2 Department of Nutrition Management, Crop Research Institute, Drnovská 507, CZ˗161 01 Prague 6, Ruzyně, Czech Republic

**Keywords:** Balanced fertilization, Enrichment factor, Heavy metal, Micronutrients, Plant-available, Soil organic matter, Soil acidification

## Abstract

One of the goals of sustainable agricultural production is to avoid soil contamination by elements defined as trace metals (TMs). The aim of this study was to assess the long-term impact of the use of pig slurry (PS) and NPK mineral fertilizers on the soil content of cadmium (Cd), copper (Cu), lead (Pb) and zinc (Zn). In a 9-year crop rotation, PS was used three times only before root crops. The same four levels of NPK doses (N_0_P_0_K_0_, N_1_P_1_K_1_, N_3_P_2_K_2_, N_4_P_2_K_2_) were applied to both plots with and without PS. Soil samples were collected in early spring from topsoil (0–0.3 m) and subsoil (0.3–0.6 m). Three forms of TMs were determined in the soil: pseudo-total (*Aqua regia)*; bioavailable (Mehlich 3 method) and readily bioavailable (mobile) forms (1 M NH_4_NO_3_). The tested factors did not have a significant impact on the Cd, Cu and Pb content, regardless of the form analyzed and the soil depth. PS application significantly increased the content of bioavailable forms of Zn regardless of the year, and the content of pseudo-total Zn only in the sugar beet year, i.e. after manure application. Increasing NPK doses increased the content of mobile Zn in the topsoil, especially in PS plots. A tendency to accumulate mobile forms of Cd and Pb was also observed on NPK-fertilized plots. Thus, long-term application of high NPK doses may increase the risk of contamination of the food chain with these metals. The content of mobile Cd and Zn was positively related to the content of total nitrogen in the soil and negatively related to pH.

**Supplementary Information:**

The online version contains supplementary material available at 10.1007/s11356-024-34993-1.

## Introduction

Soil is a key element of ecosystems related to food and feed production. Therefore, it is extremely important to recognize the long-term impact of agriculture on soil quality, especially in relation to the content of elements or chemical compounds that are non-biodegradable and accumulated in the soil over several decades or longer. These include primarily elements referred to as heavy metals (HMs). The main criterion for their selection is high atomic weight and density higher than 4.5–5 g cm^−3^ (Appenroth 2010). In addition, although their concentration in the natural environment (soil, water) is generally low, they have a significant impact on the functioning of the biosphere and human health. They can therefore also be defined as trace elements (TEs) or trace metals (TMs), depending on their chemical properties (He et al. 2005; Kabata-Pendias 2010). Among various TMs, the following elements are of particular interest: copper (Cu), zinc (Zn), cadmium (Cd) and lead (Pb). The first two TMs are essential for life and are classified as so-called micronutrients. The biochemical and physiological functions of Cu and Zn are related to their participation in the main processes occurring in the plant, namely photosynthesis, respiration, the biosynthesis of proteins, hormones, stress resistance, and the metabolism of nitrogen (N) compounds (Hänsch, and Mendel 2009). They are also essential for humans and animals (Alengebawy et al. 2021). However, they can also be potentially toxic to living organisms and may cause contamination of the food chain. An excess of Cu and Zn in the soil leads to inhibition of growth and damage to plant roots; interferes with the uptake of nutrients, especially potassium (K) and nitrogen (N); and negatively affects photosynthesis by inhibiting chlorophyll biosynthesis and the functioning of PSII (Nagajyoti et al. 2010; Chen et al. 2022). Excess Zn in the soil also negatively affects the plant-availability of P because insoluble compounds are formed (Angon et al. 2024). Furthermore, high soil concentrations of Cu can have toxic effects on microorganisms and can hinder the mineralization of organic matter (Azeez et al. 2015). In respect to animals and humans, excessive Cu and Zn intake can cause a number of problems with the functioning of digestive, immune and nervous systems, and oxidative damage due to an increase in the level of reactive oxygen species (ROS) (Mitra et al. 2022). Since excess Cu is excreted through bile, its toxicity is most likely to occur in individuals with liver diseases. Excessive Zn intake has been associated with such chronic effects as low Cu levels, including reduction of superoxide dismutase biosynthesis; impaired iron (Fe) metabolism; reduced immunity and high-density lipoprotein levels (Osredkar and Sustar 2011).

The two remaining TMs (Cd and Pb) are classified as toxic elements (Mehri and Marjan 2013). They have strong carcinogenic properties, especially Cd. They can induce cancer through various signaling pathways or by directly damaging DNA and inhibiting DNA repair systems, or through epigenetic mechanisms, e.g. abnormal DNA methylation (Wang et al. 2024). Other symptoms of excess Cd in human bodies include: nephrotoxicity, calcium metabolism alterations, osteoporosis, psychological disorders, gastrointestinal disorders, central nervous system complications, and immune system alterations. Excessive Pb absorption is responsible for: hypertension, miscarriages, renal impairment, brain injury, abdominal pain, peripheral nerve damage, encephalopathic signs, Fe deficiency due to disruption of haemoglobin synthesis, and cognitive impairment (Briffa et al. 2020). Plants respond to toxic levels of Cd and Pb by overproduction of ROS at several sites including mitochondria, chloroplasts, and peroxisomes, and at the extracellular side of the plasma membrane. They induce oxidative stress in plants, leading to a variety of damages to cellular macromolecules including lipids, proteins, and nucleic acids (Rashid et al. 2023). The secondary symptoms of excess ROS include reduced photosynthetic efficiency, plant growth rate and yield level (Alengebawy et al. 2021). In addition, excess Cd and Pb in the soil negatively affects the population size and activity of microorganisms, causing, among other things, a decrease in the rate of mineralization of organic residues, as well as the biological fixation of N_2_ (Srivastava et al. 2017).

The natural sources of TMs in soils are igneous and sedimentary rocks (Rashid et al. 2023). The sources of anthropogenic contamination of agricultural soils with TMs are: emissions from industrial and traffic sources, fertilizers and plant protection agents, and the use of sewage sludge and other waste for fertilization (Tóth et al. 2016). In intensive agricultural production, fertilizer application is a key element of proper nutrient management in the field. This factor can affect the TMs content in the soil directly or indirectly. The direct impact should be related to their specific content in organic and mineral (inorganic) fertilizers. The fertilizers from the first group are highly diversified in terms of HMs/TMs content and the potential for contamination of the food chain (Angon et al. 2024). The biggest environmental problem is posed by organic fertilizers produced from municipal and industrial wastes (sewage sludge, compost, digestate), as they contain relatively high concentrations of toxic elements, including Cd and Pb (Rashid et al. 2023). The application of organic fertilizers obtained on farms where livestock have been intentionally fed with certain TMs (Cu, Zn and even arsenic) may also pose a threat to soil quality (Wan et al. 2024). This applies in particular to slurry (Leclerc and Laurent 2017). According to the report by De Vries et al. (2022), slurry application is responsible for about 80% of the total input of Cu and Zn in European arable soils. Mineral fertilizers also differ in their TMs content and thus in their risk of soil contamination. Generally, N and K fertilizers contain trace amounts of Pb and Cd, as well as other TMs. The exceptions are N fertilizers, e.g. urea, intentionally enriched with microelements, including Cu and Zn (Babar et al. 2018). Among inorganic fertilizers, P fertilizers (superphosphates) or complex NP and NPK fertilizers based on phosphates contain the highest levels of TMs contaminants, especially Cd (Nicholson et al. 2003; Rashid et al. 2023). The application of P-containing fertilizers is responsible for 48% of the total Cd input to the soil. They are also a source of Pb in the soil. However, unlike Cd, Pb gets into the soil mainly from atmospheric deposition (45%), next from mineral fertilizers (22%) and from biosolids (15%) (De Vries et al. 2022).

The indirect effect of fertilizer application on the content of TMs in the soil is related to the impact on the chemical and biological properties of the soil, as well as the processes of mineral uptake by plants. In this context, the most important soil features include: reaction (pH), soil organic matter (SOM) content, capacity and ionic composition of the soil sorption complex, precipitation of insoluble compounds, and oxidation–reduction potential (Edmeades 2003; Cambier et al. 2014; Campillo-Cora et al. 2020; Pikuła and Stępień 2021). Among the plant-related factors, the following are important: ion antagonism and synergism, root architecture, and the mass of crop residues flowing into the soil (Keller et al. 2003). All of the above-mentioned factors affect the bioavailability of TMs in different ways, and thus the possibility of their removal from the soil in the plant biomass. There are different forms of TMs in the soil depending on their mobility and bioavailability (Feszterová et al. 2021). The most stable in the long term is the total TM content. The content of total TMs depends mainly on the balance between inflow from natural and anthropogenic sources and losses resulting either from uptake by crop plants or leaching (De Vries et al. 2022). Unlike total TMs, the content of bioavailable TMs in the soil is subject to relatively rapid changes. This form also depends to a greater extent on the processes occurring in the soil than the total content of TMs. Among the soil factors, soil reaction (pH) is particularly important (Tlustoš et al. 2006; Violante et al. 2010; Tkaczyk et al. 2017). The lower the pH, the more forms of TMs that are poorly bound to the soil, and the greater the risk of their excessive accumulation in plants (Roberts 2014; Hlisnikovský et al. 2016; Gu et al. 2022). The application of ammonium N fertilizers reduces the pH on rhizosphere and thus increases the bioavailability of TMs, including Cd (Zaccheo et al. 2006). However, the mechanism of soil acidification does not explain all the differences in the effect of fertilizers on the accumulation of TMs in plants. Some authors indicate that the presence of ammonium N in fertilizers reduces the uptake of TMs by plants compared to fertilizers containing the nitrate form (El Sharkawi and Zayed 2012). Differences in the effect of different N fertilizers are explained by the varying effects of N doses and forms on the architecture of the root system (Xue et al. 2014), uptake processes and the activity of heavy metal ATPase (HMA) family proteins (Williams and Mills 2005), and/or sink strength of the grain for some micronutrients (Zhao et al. 2009). It should also be emphasized that N fertilizers stimulate root growth and thus increase the TMs absorption potential of plants (Wang et al. 2022). Next to pH, soil organic carbon (SOC) is another important factor modifying the content of bioavailable TMs. Over a longer period of time, this factor may also affect the total content of TMs. The more SOC the soil contains, the greater its potential to adsorb, complex and chelate TMs (Campillo-Cora et al. 2020; Lan et al. 2022). Numerous studies have shown that the long-term use of animal manures, even with a low concentration of TMs, leads to their accumulation in the soil and creates a risk of over-concentration in agricultural products (Edmeades 2003; Mantovi et al. 2003; Benke et al. 2008; Qian et al. 2018). Despite numerous researches in this field, a number of questions and research problems remain unresolved. One of them is the dependence of TMs bioavailability on the interaction between pH and SOM. Roberts (2014) points out that SOM has been found to reduce Cd concentration in the soil solution in contaminated soils at pH below 6.0, but increase soil concentration at pH 6.0–8.0. Moreover, the influence of the interaction of organic and mineral fertilization on the content of TMs in the soil is poorly understood, especially in long-term crop rotations with alfalfa and cyclical use of pig slurry. The literature also lacks data on the impact of the long-term use of pig slurry on the movement of TMs between the topsoil and subsoil under conditions of simultaneous use of NPK fertilizers.

In this study, it is hypothesized that pig slurry (PS) application increases the accumulation of mobile TM fractions in the soil, and the risk is further increased under conditions of mineral NPK fertilizer application. To verify the hypothesis, the main objective was to determine the content of Cd, Cu, Pb and Zn in the soil in the pseudo-total (*Aqua regia*), bioavailable (Mehlich 3 method) and readily bioavailable (mobile, soluble in 1 M NH_4_NO_3_) forms, as a result of the long-term use of different combinations of NPK mineral fertilizers with regular pig slurry application in crop rotation with root and tuber crops. An additional objective was to determine the relationship between the concentration of bioavailable forms of TMs and basic parameters characterizing soil properties.

## Materials and methods

### Site description

The Ruzyně Fertilizer Experiment (RFE) was established on a permanent arable field in 1955. The experiment is situated in Prague-Ruzyně, Czechia (Czech Republic). Geographical coordinates are as follows: 50.088, 14.293. Altitude of the site is 370 m a.s.l.. According to many years of measurements conducted by Prague-Ruzyně meteorological station (1955–2021), the average long-term temperature in the study area is 8.2 °C (ranging from 6.4 to 9.7 °C) and the annual sum of precipitation is about 450 mm (ranging from 255 to 701 mm). The soil type is Ilimerized Luvisol developed on loess mixed with highly weathered chalk. The topsoil (0–0.3 m) is characterized by clay loam texture (27% of clay). The subsoil (0.30–0.60 m) contains about 40–49% of clay. Prior to the establishment of the experiment in 1955, the pH measured in 1 M KCl was 6.5 of the topsoil layer (0–0.2 m) and the SOC content was 1.17%.

### Experimental design

The RFE is a large-scale experiment consisting of five field strips. In this study, concentrations of TMs in soil (Cd, Cu, Pb and Zn) were determined in strip number III of the RFE named the ‘Classical Crop Rotation’(45% cereals, 33% of root crops and 22% of legumes). The crop sequence is as follows: alfalfa, alfalfa, winter wheat, sugar beet, spring barley, potatoes, winter wheat, sugar beet and spring barley with alfalfa under-sowing. In each 9-year plant rotation, pig slurry (PS) was used three times, only under tuber crops: sugar beet and potato. This fertilizer was applied in autumn after the harvest of the forecrop (winter wheat and spring barley). Rates of the PS depended on the plant species and amounted to 68 t ha^−1^ for sugar beet and 49 t ha^−1^ for potato. The chemical composition of PS is presented in Table 1. Depending on the root crop cultivated and the PS dose applied, the nitrogen (N) supply to the soil during a single crop rotation cycle was 64–89 kg ha^−1^; phosphorus (P) 28–39 kg ha^−1^, and potassium (K) 63–88 kg ha^−1^. Mineral fertilizers were used at 4 levels of varied nitrogen balance, regardless of the variant of PS application (N_0_P_0_K_0_; N_1_P_1_K_1_; N_3_P_2_K_2_ and N_4_P_2_K_2_). As a result, the total number of fertilizer combinations was 8 (Table 2). Calcium ammonium nitrate (27% N), single superphosphate (8.3% P) and potassium chloride (49.8% K) were applied as mineral fertilizers. The rates of mineral fertilizer doses depended not only on the degree of N balance, but also on the crop. The detailed annual rates of the macronutrients (NPK) in mineral fertilizers and PS for each plant separately in the one crop rotation is presented in the supplementary material (Table S1). Mean annual inputs of NPK in mineral fertilizers and PS over one 9-year crop rotation are presented in Table 2. As the table shows, in the treatments with PS, the average total NPK income on the plots was higher than in the treatments with mineral fertilizers only.

**Table 1:** Chemical composition of pig slurry used for potatoes and sugar beet

Characteristics	Unit	Range	Mean
pH	-	6.7–7.4	7.1
Dry matter (DM)	g kg^−1^	42.3–60.6	51.4
Ash	g kg^−1^ of DM	166.8–226.3	196.5
Conductivity	mS m^−1^	2377–2441	2409
T^*^N	g kg^−1^ of DM	25.1–26.0	25.5
TP	g kg^−1^ of DM	9.8–12.5	11.1
TK	g kg^−1^ of DM	23.2–26.9	25.1
TCa	g kg^−1^ of DM	21.0–25.7	23.3
TMg	g kg^−1^ of DM	7.0–9.7	8.3
TCu	mg kg^−1^ of DM	136.1–159.7	147.9
TZn	mg kg^−1^ of DM	384.5–657.6	521.0
TPb	mg kg^−1^ of DM	1.09–1.60	1.35
TCd	mg kg^−1^ of DM	0.276–0.477	0.376

^*^T total content

**Table 2:** Mean annual inputs of the main macronutrients (NPK) and trace metals (Cd, Cu, Pb and Zn) in mineral fertilizers and pig slurry over one 9-year crop rotation

Treatments	Macronutrients kg ha^−1^			Trace metals g ha^−1^			
	N	P	K	Cd	Cu	Pb	Zn
N_0_P_0_K_0_	0	0	0	0	0	0	0
N_1_P_1_K_1_	39	24	109	0.40	2.6	0.43	32.2
N_3_P_2_K_2_	67	31	146	0.52	3.4	0.59	42.2
N_4_P_2_K_2_	91	31	146	0.52	3.5	0.64	42.7
PS + N_0_P_0_K_0_	27	12	27	0.40	156.3	1.43	550.4
PS + N_1_P_1_K_1_	66	36	135	0.80	158.8	1.85	582.7
PS + N_3_P_2_K_2_	94	43	173	0.92	159.7	2.02	592.7
PS + N_4_P_2_K_2_	116	43	173	0.92	159.8	2.06	593.1

The input of TMs to the soil in PS was calculated on the basis of its chemical composition from two recent years, 2019 and 2020 (Table 1). In the years of PS application TM doses were as follows: Cd 1.0–1.3, Cu 373–517, Pb 3.0–4.7 and Zn 1312–1821 g ha^−1^, depending on the tuber crop and slurry dose. The input of TMs to the soil in mineral fertilizers was calculated taking into account their content in applied fertilizers: calcium ammonium nitrate (27% N), single superphosphate (8.3% P) and potassium chloride (49.8% K). The concentration of Cd, Cu, Pb and Zn in calcium ammonium nitrate was as follows: 0.02, 1.46, 0.54, 5.84 mg kg^−1^_,_ respectively. For superphosphate it was 1.37, 7.82, 0.73 and 105.7, and for potassium salts 0.02, 0.49, 0.63 and 4.44 mg kg^−1^, for Cd, Cu, Pb and Zn respectively. The content of TMs in PS and NPK fertilizers was determined in the chemical laboratory in Chomutov, belonging to the Crop Research Institute (Prague, Czechia). The presented data indicate that P fertilizers were the main source of TMs in the soil, in particular Cd and Zn. The average annual inflow of TMs to the soil in one crop rotation, both from mineral NPK fertilisers and PS, is shown in Table 2.

### Soil sampling and analysis

Soil samples for the assessment of TM content were collected in early spring, before fertilizer application and the start of vegetation, in two growing seasons: 2020 and 2021. Soil samples were taken from two layers: topsoil (0–0.3 m) and subsoil (0.3–0.6 m). Soil samples were collected randomly from each plot using a stainless steel soil sampler probe. The total number of bulk soil samples amounted to 64. The bulk soil samples were air dried at room temperature (18–20 ºC), then ground in a porcelain mortar, and sieved to 2 mm.

The soil pH was measured in a solution of 1 M KCl (soil/solution ratio was 1:2.5, w/v). Plant-available macronutrient (P, K, Mg and Ca) concentrations were determined by the Mehlich 3 method (Mehlich 1984). P concentration in the extracts was measured by using the ascorbic-ammonium molybdate method. The intensity of blue coloration was measured using a UV–VIS spectrophotometer (Jasco V-630, Jasco Internatiol Co, LTD, Tokyo, Japan) at a 800-nm wavelength. The concentrations of basic cations (K, Mg and Ca) in the Mehlich 3 solution were analysed by the Atomic Absorption Spectrometry (AAS) method (ThermoScientific iCE 3000 Series, Thermo Fisher Scientific Inc., Waltham, USA). Total nitrogen (TN) content was determined by the Kjeldahl method using a thermal block and a distillation unit (FOSS, Denmark). The total carbon content in the soil (TSC) was determined using a carbon/sulphur analyzer ELTRA CS–2000 (ELTRA GmbH, Haan, Germany). Soil carbonate was determined by acid dissolution followed by the volumetric analysis of the released carbon dioxide (CO_2_). The Scheibler apparatus was used for this purpose. Since the carbonate content was trace, no data was presented, and it was assumed that TSC was mainly organic C (SOC).

Three forms of TMs were determined in the soil: i) pseudo-total content was measured using standard *Aqua regia* (abbreviation T); ii) bioavailable (plant-available) forms by using the Mehlich 3 method (M3), and iii) mobile forms (labile, readily bioavailable forms to plants) by using ammonium nitrate, NH_4_NO_3_ (L). In order to determine the pseudo-total content of TMs, the soil samples were additionally ground to powder (< 0.15 mm) in an agate mortar. Next, a soil sample of 2.0 g was placed in a glass tube with the cap (the unheated part of the tube served as a condenser) and digested in 20 mL of *Aqua regia* in a heating block at 105 °C for 2 h, followed by filtration (ISO 54321 2020). The filtrate was filled up with deionized water to the 50 mL mark. The plant-available forms of TMs were obtained using a standard Mehlich 3 procedure in the Czechia (Malý et al. 2021). In order to obtain mobile forms of TMs, 25 mL of 1.0 M NH_4_NO_3_ solution was added to 10 g of soil, after which the suspension was shaken for 2 h (Gryschko et al. 2005). The soil extracts were centrifuged to obtain a clear solution at 3000 rpm lasting 5 min. Concentrations of TMs in soil digests and extracts were determined by Atomic Absorption Spectrometry (AAS) using a ThermoScientific iCE 3000 Series (Thermo Fisher Scientific Inc., Waltham, USA). All reagents were of analytical grade. Standard stock solutions of metals (such as nitrate) were obtained from Merck (Darmstadt, Germany), and other reagents were purchased from Chempur (Piekary Śląskie, Poland).

### Percentage of bioavailable forms and topsoil enrichment factor

The bioavailable forms of TMs in soils as a percentage of the pseudo-total content were calculated according to the following algorithms:

1 $$\text{M}3/\text{T}=\left(\text{M}3\times 100\right)/\text{ T}$$

2 $$\text{L}/\text{T}=\left(\text{L}\times 100\right)/\text{ T}$$

where, M3/T – proportion of plant-available metals in the pseudo-total content, %; L/T – proportion of mobile metals in the pseudo-total content, %; T – content of pseudo-total (*Aqua regia*) metals, mg kg^−1^; M3 – content of plant-available (Mehlich 3) metals, mg kg^−1^; L – content of mobile (1 M NH_4_NO_3_) metals, mg kg^−1^.

The topsoil enrichment factor (TEF), relative to subsoil, was calculated as the ratio of the TM content in topsoil and subsoil:

3 $$\text{TEF}={\text{C}}_{\text{top}}/{\text{C}}_{\text{sub}}$$

where TEF is the topsoil enrichment factor; C_top_ is the content of metals in the topsoil (0–0.3 m), mg kg^−1^; C_sub_ is the content of metals in the subsoil (0.3–0.6 m), mg kg^−1^. The TEF coefficient was calculated for each element and its forms of TMs in the soil.

### Statistical analysis

The effects of individual research factors (year, fertilization treatments) and their interactions were assessed by means of the two-way ANOVA, separately for topsoil and subsoil. Means were separated by honest significant difference (HSD) using Tukey’s method. The distribution of the data (normality) was checked using the Shapiro–Wilk test and the homogeneity of variance by the Bartlett test. Standard error of the mean (SEM) was used to indicate statistical error. Pearson’s correlation and linear regression was applied for the evaluation of the relationships between variables. Statistica 13 software was used for all statistical analyses (TIBCO Software Inc. 2020).

## Results

### Basic soil chemical properties

The basic chemical properties of the soil are presented in Table 3. The pH of the soils in the arable layer was acidic. As a result of many years of fertilization, the pH had decreased by more than 1 unit compared to 1955. Slurry application, regardless of the soil depth, led to an increase in soil acidity. On average, the pH of the soil in the tillage layer (topsoil) for the treatments without PS was 5.36 and with it 5.14. For the subsoil layer the results were 5.91 and 5.46, respectively. Increasing levels of mineral fertilization also lowered the pH, especially in soil samples collected from plots fertilized by PS. On the other hand, in plots without PS, the highest pH was recorded in the N_1_P_1_K_1_ treatment, regardless of the soil depth. Higher rates of NPK reduced the pH. The application of PS increased the mean content of TSC, TN, plant-available forms of P, K and Mg in topsoil, while it led to a decrease in Ca content. A trend towards an increase in TSC and TN was observed in soil samples collected from plots fertilized with higher doses of NPK. Increasing rates of P and K improved the abundance of both elements in the topsoil. At the same time, higher doses of NPK led to a decrease in the level of Mg and Ca content in the arable layer. From the point of view of soil fertility, PS-N_3_P_2_K_2_ can be considered the optimal treatment, in which not only the highest content of TSC, TN, P and K was obtained, but also a slight reduction in the concentration of Mg. Fertilization had no significant effect on the TSC, TN and C:N ratio in the subsoil. However, a trend could be observed indicating that NPK doses had a greater effect on TSC than the use of PS. Moreover, samples collected from plots fertilized with PS were characterized by a narrower C:N ratio. As the input of P and K into the soil with fertilizers increased, the content of M3P and M3K also increased. In turn, in plots without PS, increasing doses of NPK decreased the magnesium content in the subsoil (Table 3).

**Table 3:** Basic chemical properties of soil after long-term use of pig slurry (PS) and different doses of NPK mineral fertilizers (mean ± SEM for years 2020–2021)

Treatments	pH	TSC %	TN %	C:N	M3P mg kg^−1^	M3K mg kg^−1^	M3Ca mg kg^−1^	M3Mg mg kg^−1^
Topsoil (0.0–0.3 m)								
N_0_P_0_K_0_	5.35 ± 0.09^ab^	1.34 ± 0.12^c^	0.120 ± 0.01^d^	11.1 ± 0.87	43 ± 17.0^d^	162 ± 16.8^d^	1841 ± 164	206 ± 25.5^ab^
N_1_P_1_K_1_	5.53 ± 0.55^a^	1.37 ± 0.07^abc^	0.120 ± 0.01^d^	11.5 ± 0.63	87 ± 33.8^c^	205 ± 11.5^c^	1987 ± 520	193 ± 37.9^ab^
N_3_P_2_K_2_	5.40 ± 0.44^ab^	1.36 ± 0.07^bc^	0.125 ± 0.00^cd^	10.8 ± 0.76	115 ± 21.0^b^	221 ± 22.5^abc^	1758 ± 339	178 ± 37.1^b^
N_4_P_2_K_2_	5.16 ± 0.48^ab^	1.35 ± 0.11^bc^	0.128 ± 0.02^bcd^	10.5 ± 0.72	104 ± 36.6^bc^	212 ± 11.7^c^	1681 ± 438	179 ± 40.6^b^
PS + N_0_P_0_K_0_	5.40 ± 0.31^ab^	1.39 ± 0.06^abc^	0.125 ± 0.00^ cd^	11.3 ± 1.50	86 ± 24.4^c^	170 ± 15.4^d^	1803 ± 171	221 ± 26.3^a^
PS + N_1_P_1_K_1_	5.26 ± 0.37^ab^	1.43 ± 0.06^abc^	0.133 ± 0.00^abc^	10.7 ± 0.22	145 ± 26.5^a^	213 ± 10.5^bc^	1677 ± 181	202 ± 29.1^ab^
PS + N_3_P_2_K_2_	5.06 ± 0.28^ab^	1.49 ± 0.05^a^	0.139 ± 0.01^ab^	10.7 ± 0.29	173 ± 14.9^a^	243 ± 14.2^a^	1582 ± 237	197 ± 28.1^ab^
PS + N_4_P_2_K_2_	4.86 ± 0.19^b^	1.46 ± 0.05^ab^	0.141 ± 0.00^a^	10.4 ± 0.33	164 ± 24.9^a^	238 ± 17.8^ab^	1517 ± 251	194 ± 32.4^ab^
Subsoil (0.3–0.6 m)								
N_0_P_0_K_0_	5.97 ± 0.53^ab^	0.95 ± 0.20	0.09 ± 0.01	10.9 ± 0.99	24 ± 11.4^c^	162 ± 15.9^b^	2826 ± 778	247 ± 25.3^a^
N_1_P_1_K_1_	6.28 ± 0.65^a^	0.99 ± 0.25	0.09 ± 0.01	11.2 ± 2.57	44 ± 14.6^bc^	174 ± 24.0^ab^	3113 ± 947	235 ± 16.9^ab^
N_3_P_2_K_2_	5.79 ± 0.66^ab^	0.99 ± 0.20	0.09 ± 0.01	10.9 ± 1.99	46 ± 13.7^bc^	171 ± 30.2^ab^	2622 ± 959	213 ± 43.7^ab^
N_4_P_2_K_2_	5.61 ± 0.79^ab^	1.16 ± 0.37	0.10 ± 0.01	11.9 ± 3.99	58 ± 15.5^ab^	180 ± 40.6^ab^	2465 ± 829	201 ± 33.7^b^
PS + N_0_P_0_K_0_	5.48 ± 0.23^ab^	0.96 ± 0.14	0.10 ± 0.01	10.0 ± 0.56	42 ± 9.7^bc^	174 ± 10.1^ab^	2356 ± 248	244 ± 15.0^a^
PS + N_1_P_1_K_1_	5.54 ± 0.30^ab^	0.89 ± 0.11	0.09 ± 0.01	9.8 ± 0.51	52 ± 16.4^ab^	184 ± 9.1^ab^	2410 ± 110	237 ± 18.3^ab^
PS + N_3_P_2_K_2_	5.37 ± 0.34^b^	0.98 ± 0. 14	0.10 ± 0.01	10.1 ± 0.86	76 ± 19.8^a^	194 ± 15.7^a^	2413 ± 228	240 ± 17.8^ab^
PS + N_4_P_2_K_2_	5.44 ± 0.48^ab^	1.01 ± 0 .19	0.10 ± 0.02	9.9 ± 0.56	77 ± 27.9^a^	194 ± 15.7^a^	2439 ± 436	243 ± 25.3^ab^

Key: *pH* soil reaction in 1 M KCl, *TSC* total soil carbon, *TN* total nitrogen, *C:N – TSC* TN ratio, M3P, M3K, M3Ca and M3Mg – plant available forms determined by Mehlich 3 method. Different letters indicate statistically significant differences between treatments at *p* ≤ 0.05 (HSD test)

### Pseudo-total content of metals

The experiment showed a slightly higher pseudo-total TM content in topsoil (TZn – 66.5, TCu – 28.7, TPb – 22.1 and TCd – 0.37 mg kg^−1^) compared to the subsoil (TZn – 65.4, TCu – 28.5, TPb – 19.8 and TCd – 0.31 mg kg^−1^). The growing season did not significantly differentiate the content of pseudo-total TM content in the soil, regardless of the metal and soil depth (Table S2). Long-term mineral-organic fertilization also had no significant impact on the content of TMs. However, a trend towards an increase in TZn content was observed in soil samples cyclically fertilized with PS (Table 4). Soil samples collected in spring 2021 even showed a significant impact of PS application on the TZn content. It was the growing season in which sugar beets were grown and, according to the experimental scheme, pig slurry was applied in autumn 2020. A clear trend towards an increase in the pseudo-total Zn in the topsoil as a result of the use of PS was also observed in spring 2020 (Table S3). As a result, regardless of NPK doses, the average increase of TZn content in topsoil following long-term PS use was 10.2%. The slurry application also increased the average concentration of TZn in the subsoil by 7.2%. The doses of mineral fertilizers did not have a significant impact on the TZn content in the topsoil and subsoil. However, of all the treatments, the one with PS and the highest dose of NPK (PS + N_4_P_2_K_2_) had the greatest effect on the average TZn content in topsoil. On the other hand, the lowest concentration of TZn was obtained in the N_1_P_1_K_1_ treatment. The experiment also revealed a slight increase in TCu and TPb content as a result of using PS, especially in subsoil. In addition, there was a trend towards an increase in the TCd content in topsoil and subsoil as a result of long-term PS and NPK fertilization. The lowest concentration of this element was obtained in the treatment without any fertilizers (N_0_P_0_K_0_).

**Table 4:** Effect of long-term use of pig slurry (PS) and different doses of NPK mineral fertilizers on the content of pseudo-total (*Aqua regia*) trace metals (mean ± SEM for the years 2020–2021)

Treatments	TCd	TCu	TPb	TZn
	mg kg^−1^			
Topsoil (0.0–0.3 m)				
N_0_P_0_K_0_	0.30 ± 0.05	28.9 ± 1.04	23.2 ± 2.93	63.1 ± 1.73
N_1_P_1_K_1_	0.41 ± 0.06	28.7 ± 1.06	23.6 ± 1.80	62.1 ± 2.53
N_3_P_2_K_2_	0.43 ± 0.09	27.9 ± 0.94	21.4 ± 2.70	63.8 ± 1.99
N_4_P_2_K_2_	0.33 ± 0.06	28.9 ± 1.60	20.8 ± 2.30	64.2 ± 3.57
PS + N_0_P_0_K_0_	0.42 ± 0.11	29.4 ± 1.12	22.3 ± 1.63	70.2 ± 2.95
PS + N_1_P_1_K_1_	0.33 ± 0.06	29.4 ± 0.99	22.1 ± 1.48	68.1 ± 2.00
PS + N_3_P_2_K_2_	0.36 ± 0.04	27.1 ± 0.75	22.4 ± 1.87	69.4 ± 2.50
PS + N_4_P_2_K_2_	0.41 ± 0.06	29.5 ± 0.86	21.3 ± 0.97	71.4 ± 4.20
Subsoil (0.3–0.6 m)				
N_0_P_0_K_0_	0.26 ± 0.06	28.4 ± 1.77	19.3 ± 2.81	64.1 ± 2.00
N_1_P_1_K_1_	0.33 ± 0.05	27.4 ± 1.04	18.5 ± 3.61	62.9 ± 0.95
N_3_P_2_K_2_	0.34 ± 0.05	27.8 ± 1.64	17.6 ± 3.79	63.4 ± 1.78
N_4_P_2_K_2_	0.30 ± 0.06	27.9 ± 1.13	19.6 ± 2.85	61.9 ± 1.29
PS + N_0_P_0_K_0_	0.32 ± 0.06	28.1 ± 1.20	20.5 ± 2.10	70.3 ± 4.67
PS + N_1_P_1_K_1_	0.27 ± 0.04	29.2 ± 2.07	19.9 ± 2.97	66.9 ± 4.15
PS + N_3_P_2_K_2_	0.28 ± 0.08	28.7 ± 1.49	20.1 ± 2.68	66.2 ± 1.69
PS + N_4_P_2_K_2_	0.36 ± 0.10	30.6 ± 1.50	22.6 ± 3.76	69.8 ± 2.23

### Plant-available content of metals

In our study, in contrast to the pseudo-total forms of TMs, the content of plant-available forms of Cu, Pb and Zn (M3Cu, M3Pb and M3Zn) in the topsoil depended significantly on the growing season (Table S2, S4). The average content of these metals was higher in 2021 (M3Zn – 5.84, M3Cu – 5.93 and M3Pb – 6.51 mg kg^−1^) than 2020 (M3Zn – 3.75, M3Cu – 4.50 and M3Pb – 6.14 mg kg^−1^). These differences can be explained by the use of slurry in the 2020/2021 growing season and/or soil factors increasing the pool of more mobile forms of elements. In addition, in contrast to the pseudo-total form of TMs, significantly more M3 metals accumulated in the topsoil than in the subsoil. The differences, depending on the element and the year of study, were 36.3–72.6%, 25.3–36.0%, 43.9–51.9% and 13.1–18.7%, respectively to M3Zn, M3Cu, M3Pb and M3Cd. Among the analysed elements, only the M3Zn content significantly depended on the fertilizer factor. In soil samples collected from plots fertilised with PS alone or together with NPK fertilisers (PS; PS + N_1_P_1_K_1_; PS + N_3_P_2_K_2_) more M3Zn was found than in soil samples from treatments: N_0_P_0_K_0_; N_1_P_1_K_1_; N_4_P_2_K_2_ (Table 5). The difference was approximately 60%. Regardless of mineral NPK fertilization, the application of PS increased the average concentration of M3Zn by 55.7% in the topsoil and 26.9% in the subsoil. PS application also resulted in an increase in the M3Cu content compared to plots without PS. The average difference was 18.7% in topsoil and 10.6% in subsoil. In contrast to M3Zn and M3Cu, the contents of M3Cd and M3Pb were only slightly affected by PS fertilization.

**Table 5:** Effect of long-term use of pig slurry (PS) and different doses of NPK mineral fertilizers on the content of plant-available (Mehlich 3) trace metals (mean ± SEM for the years 2020–2021)

Treatments	M3Cd	M3Cu	M3Pb	M3Zn
	mg kg^−1^			
Topsoil (0.0–0.3 m)				
N_0_P_0_K_0_	0.19 ± 0.01	4.81 ± 0.52	6.33 ± 0.63	3.67 ± 0.55^b^
N_1_P_1_K_1_	0.18 ± 0.01	4.78 ± 0.52	6.74 ± 0.52	3.71 ± 0.52^b^
N_3_P_2_K_2_	0.18 ± 0.02	5.01 ± 0.42	7.42 ± 0.87	3.94 ± 0.42^ab^
N_4_P_2_K_2_	0.18 ± 0.02	4.48 ± 0.58	6.39 ± 0.86	3.66 ± 0.27^b^
PS + N_0_P_0_K_0_	0.18 ± 0.01	6.01 ± 0.32	7.50 ± 0.25	6.04 ± 0.58^a^
PS + N_1_P_1_K_1_	0.18 ± 0.01	5.73 ± 0.45	6.77 ± 0.46	5.96 ± 0.70^a^
PS + N_3_P_2_K_2_	0.19 ± 0.01	5.62 ± 0.52	6.78 ± 0.57	6.10 ± 0.76^a^
PS + N_4_P_2_K_2_	0.19 ± 0.01	5.26 ± 0.43	6.65 ± 0.49	5.26 ± 0.69^ab^
Subsoil (0.3–0.6 m)				
N_0_P_0_K_0_	0.15 ± 0.01	3.52 ± 0.48	4.22 ± 0.53	2.54 ± 0.49
N_1_P_1_K_1_	0.15 ± 0.01	4.09 ± 0.36	4.99 ± 0.46	3.03 ± 0.41
N_3_P_2_K_2_	0.16 ± 0.01	3.65 ± 0.47	4.52 ± 0.45	2.71 ± 0.44
N_4_P_2_K_2_	0.16 ± 0.01	4.00 ± 0.54	5.37 ± 0.57	3.10 ± 0.47
PS + N_0_P_0_K_0_	0.15 ± 0.01	4.37 ± 0.56	4.58 ± 0.42	3.65 ± 0.70
PS + N_1_P_1_K_1_	0.15 ± 0.01	3.90 ± 0.32	4.02 ± 0.30	3.32 ± 0.40
PS + N_3_P_2_K_2_	0.16 ± 0.01	4.14 ± 0.36	4.60 ± 0.43	3.69 ± 0.51
PS + N_4_P_2_K_2_	0.17 ± 0.01	4.48 ± 0.52	4.83 ± 0.55	3.78 ± 0.60

Different letters indicate statistically significant differences between treatments at *p* ≤ 0.05 (HSD test)

### Content of metals in mobile form

The mean content of Zn extracted in a solution of 1 M NH_4_NO_3_ (LZn) was 0.36 mg kg^−1^ in the topsoil and 0.12 mg kg^−1^ in subsoil. For LPb 0.24 and 0.23 were obtained, for LCu 0.076 and 0.059, and for LCd 0.096 and 0.091 mg kg^−1^, respectively to soil depth. In a similar way to the plant-available form of TMs, the content of LZn, LPb and LCd in topsoil depended on the growing season (Table S2, S5). Higher values were obtained in 2021. Of all the analysed metals, only the content of LZn was significantly affected by the fertilizer factor (Table 6). At the same time, this impact was independent of the growing season. On average, the highest concentration of LZn was found in the PS + N_4_P_2_K_2_ treatment. Significantly lower values were obtained in the N_0_P_0_K_0_, N_1_P_1_K_1_ and N_3_P_2_K_2_ treatments. On average, PS application increased the LZn content in topsoil by 78.1% and in subsoil by 67.0%. Long-term application of slurry and NPK doses did not have a significant effect on the accumulation of LCu and LPb in the soil. With regard to LCd, a trend towards an increase in the content of this element was observed as a result of the application of slurry.

**Table 6:** Effect of long-term use of pig slurry (PS) and different doses of NPK mineral fertilizers on the content of mobile (1 M NH_4_NO_3_) trace metals (mean ± SEM for the years 2020–2021)

Treatments	LCd	LCu	LPb	LZn
	mg kg^−1^			
Topsoil (0.0–0.3 m)				
N_0_P_0_K_0_	0.09 ± 0.01	0.09 ± 0.03	0.18 ± 0.03	0.26 ± 0.03^c^
N_1_P_1_K_1_	0.09 ± 0.01	0.07 ± 0.01	0.24 ± 0.05	0.23 ± 0.05^c^
N_3_P_2_K_2_	0.09 ± 0.01	0.07 ± 0.01	0.28 ± 0.03	0.22 ± 0.05^c^
N_4_P_2_K_2_	0.10 ± 0.01	0.07 ± 0.01	0.27 ± 0.04	0.33 ± 0.07^bc^
PS + N_0_P_0_K_0_	0.09 ± 0.01	0.08 ± 0.01	0.22 ± 0.05	0.29 ± 0.06^bc^
PS + N_1_P_1_K_1_	0.10 ± 0.01	0.09 ± 0.02	0.22 ± 0.05	0.46 ± 0.08^bc^
PS + N_3_P_2_K_2_	0.10 ± 0.01	0.06 ± 0.01	0.26 ± 0.02	0.55 ± 0.08^ab^
PS + N_4_P_2_K_2_	0.11 ± 0.01	0.08 ± 0.01	0.26 ± 0.03	0.56 ± 0.10^a^
Subsoil (0.3–0.6 m)				
N_0_P_0_K_0_	0.08 ± 0.01	0.08 ± 0.02	0.23 ± 0.04	0.08 ± 0.02
N_1_P_1_K_1_	0.08 ± 0.01	0.07 ± 0.01	0.21 ± 0.06	0.07 ± 0.03
N_3_P_2_K_2_	0.09 ± 0.01	0.07 ± 0.01	0.26 ± 0.04	0.08 ± 0.03
N_4_P_2_K_2_	0.09 ± 0.01	0.06 ± 0.01	0.26 ± 0.04	0.13 ± 0.04
PS + N_0_P_0_K_0_	0.09 ± 0.01	0.05 ± 0.01	0.21 ± 0.05	0.14 ± 0.04
PS + N_1_P_1_K_1_	0.10 ± 0.01	0.04 ± 0.01	0.20 ± 0.04	0.12 ± 0.03
PS + N_3_P_2_K_2_	0.10 ± 0.01	0.05 ± 0.01	0.22 ± 0.03	0.19 ± 0.07
PS + N_4_P_2_K_2_	0.10 ± 0.01	0.06 ± 0.01	0.25 ± 0.05	0.14 ± 0.05

Different letters indicate statistically significant differences between treatments at *p* ≤ 0.05 (HSD test)

### Percentage of bioavailable forms and topsoil enrichment factor

The average proportion of metals soluble in Mehlich's 3 reagent in their pseudo-total content (M3/T) was 7.1, 18.2, 31.1 and 50.5% in topsoil, for Zn, Cu, Pb and Cd respectively. For subsoil 5.0, 14.5, 26.8 and 54.7% were found. Fertilization did not exert a significant effect on the proportion of plant-available Cd, Cu and Pb in their pseudo-total content (Table 7). A significant effect of fertilization was obtained only for Zn, and in topsoil. As a result of PS use, the proportion of M3Zn in TZn increased, especially in the treatments: PS, PS + N_1_P_1_K_1_ and PS + N_3_P_2_K_2_. The proportion of labile forms in the pseudo-total metal content (L/T) was lower than for the forms determined by the Mehlich 3 method, especially for LCu, LZn and LPb. For comparison, 0.27 and 0.21% were obtained for LCu, 0.54 and 0.18% for LZn, and 1.21 and 1.52% for LPb, respective to soil depth. The ratio of LCd/TCd was higher than for the previous metals; in topsoil it was 26.6% and in subsoil 32.6%. Fertilization significantly influenced only the proportion of LZn in TZn in topsoil. The highest ratio of LZn/TZn was obtained in treatment with PS and the highest dose of NPK (Table 7).

**Table 7:** Proportion of bioavailable forms in pseudo-total (*Aqua regia*) trace metal content depending on the soil depth and long-term use of pig slurry (PS) and different doses of NPK mineral fertilizers (mean ± SEM for the years 2020–2021)

Treatments	M3/T proportion (%)					L/T proportion (%)						
	Cd	Cu	Pb		Zn	Cd		Cu		Pb		Zn
Topsoil (0.0–0.3 m)												
N_0_P_0_K_0_	64.2 ± 15.2	16.8 ± 1.9	28.3 ± 4.2		5.78 ± 0.85^b^	32.2 ± 10.4		0.29 ± 0.10		0.77 ± 0.18		0.41 ± 0.05^bc^
N_1_P_1_K_1_	43.5 ± 3.8	16.7 ± 1.8	28.9 ± 4.5		5.91 ± 0.73^b^	22.0 ± 0.6		0.24 ± 0.04		1.06 ± 0.25		0.36 ± 0.08^c^
N_3_P_2_K_2_	43.3 ± 5.9	18.0 ± 1.7	36.0 ± 10.5		6.15 ± 0.62^b^	21.5 ± 0.7		0.26 ± 0.05		1.30 ± 0.26		0.35 ± 0.07^c^
N_4_P_2_K_2_	54.0 ± 6.3	15.7 ± 2.2	29.3 ± 5.3		5.76 ± 0.96^b^	29.7 ± 4.9		0.26 ± 0.06		1.31 ± 0.33		0.52 ± 0.12^bc^
PS + N_0_P_0_K_0_	43.8 ± 0.4	20.4 ± 1.2	33.7 ± 2.7		8.58 ± 0.78^a^	22.6 ± 2.7		0.26 ± 0.04		1.02 ± 0.27		0.41 ± 0.08^bc^
PS + N_1_P_1_K_1_	55.1 ± 4.6	19.6 ± 1.7	31.1 ± 3.3		8.68 ± 1.03^a^	30.0 ± 2.5		0.31 ± 0.06		1.04 ± 0.28		0.66 ± 0.11^bc^
PS + N_3_P_2_K_2_	52.5 ± 7.1	20.6 ± 2.0	30.4 ± 3.6		8.74 ± 1.09^a^	28.0 ± 2.1		0.24 ± 0.04		1.15 ± 0.14		0.78 ± 0.11^ab^
PS + N_4_P_2_K_2_	47.7 ± 6.3	17.9 ± 1.5	31.3 ± 2.6		7.35 ± 1.12^ab^	26.7 ± 3.5		0.27 ± 0.03		1.22 ± 0.17		0.80 ± 0.16^a^
Subsoil (0.3–0.6 m)												
N_0_P_0_K_0_	72.2 ± 31.9	12.8 ± 2.0	24.3 ± 5.4		3.94 ± 0.77	42.6 ± 23.4		0.27 ± 0.05		1.32 ± 0.42		0.12 ± 0.03
N_1_P_1_K_1_	48.3 ± 7.7	15.2 ± 1.6	30.7 ± 8.0		4.79 ± 0.60	26.3 ± 7.6		0.26 ± 0.05		1.36 ± 0.67		0.11 ± 0.04
N_3_P_2_K_2_	47.8 ± 3.5	13.5 ± 1.9	28.2 ± 11.0		4.26 ± 0.66	26.0 ± 4.2		0.25 ± 0.05		1.69 ± 0.49		0.12 ± 0.04
N_4_P_2_K_2_	60.8 ± 19.9	14.8 ± 2.4	30.2 ± 4.9		4.98 ± 0.73	36.7 ± 16.2		0.23 ± 0.03		1.47 ± 0.31		0.21 ± 0.07
PS + N_0_P_0_K_0_	47.5 ± 1.4	15.9 ± 2.2	25.0 ± 4.6		5.33 ± 1.08	29.0 ± 3.6		0.17 ± 0.04		1.22 ± 0.35		0.21 ± 0.07
PS + N_1_P_1_K_1_	56.7 ± 4.7	14.1 ± 2.0	23.9 ± 4.2		5.36 ± 1.05	37.4 ± 8.7		0.12 ± 0.04		1.39 ± 0.38		0.19 ± 0.09
PS + N_3_P_2_K_2_	57.9 ± 10.6	14.8 ± 1.3	25.7 ± 4.7		5.58 ± 0.71	36.5 ± 10.2		0.17 ± 0.03		1.26 ± 0.29		0.28 ± 0.10
PS + N_4_P_2_K_2_	46.2 ± 2.1	15.0 ± 1.8	26.0 ± 5.6		5.46 ± 0.88	27.0 ± 1.6		0.20 ± 0.05		1.41 ± 0.66		0.21 ± 0.07

Different letters indicate statistically significant differences between treatments at *p* ≤ 0.05 (HSD test)

**Fig. 1:**
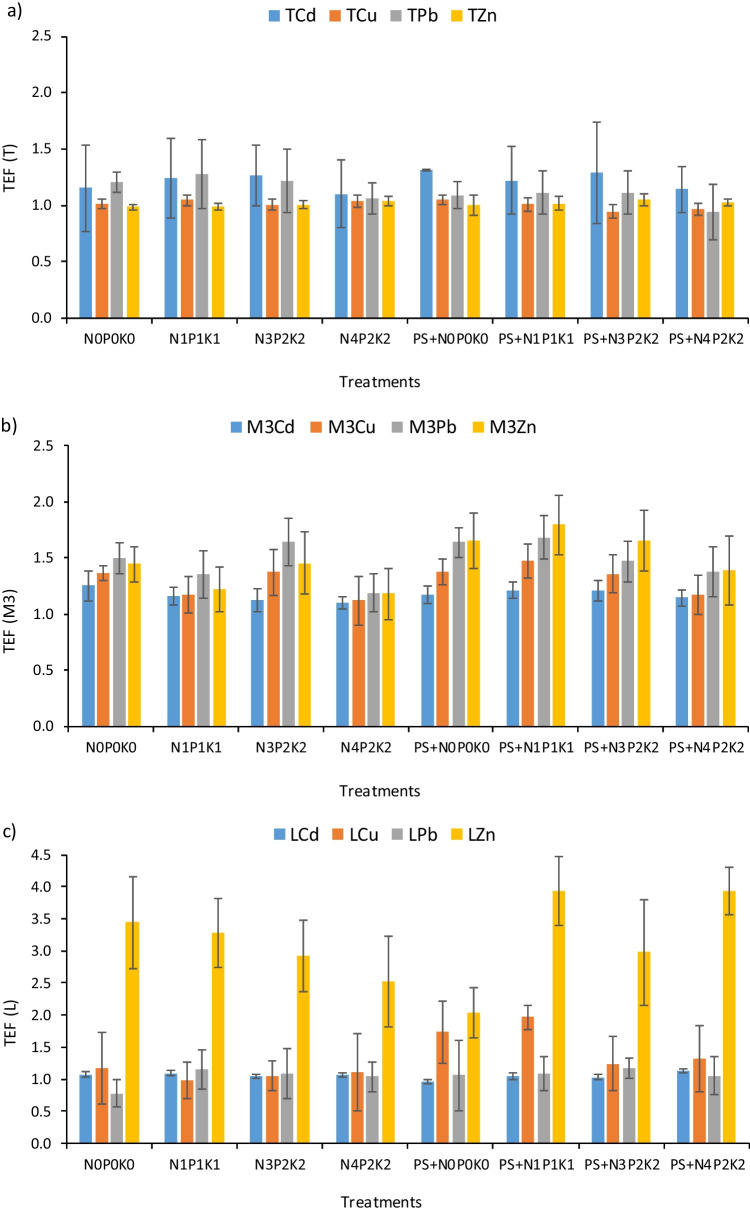
Effect of long-term use of pig slurry (PS) and different doses of NPK mineral fertilizers on topsoil enrichment factor (TEF) for: **a** T – pseudo-total (*Aqua Regia*); **b** M3 – plant-available (Mehlich3); **c** L – mobile (1 M NH_4_NO_3_) trace metals

The topsoil enrichment factor (TEF) was on average 1.00, 1.01, 1.12 and 1.22, respectively for TCu, TZn, TPb and TCd. This indicates a slight enrichment of topsoil in the tested metals. In addition, the fertilization treatments did not have a significant impact on the TEF of the pseudo-total TMs (Fig. 1). There was only a trend towards lower TEF values for TCd and TPb in the treatments with the highest dose of NPK, both in plots with and without PS. TEF factors for M3 forms were higher than for the pseudo-total forms. They averaged 1.17, 1.30, 1.47 and 1.48, respectively for M3Cd, M3Cu, M3Zn and M3Pb. The highest TEF values for M3Zn and M3Cu were found in the PS treatments, especially in PS + N_1_P_1_K_1_. Higher doses of NPK fertilizers reduced the TEF coefficient for these metals. However, the differences compared to the control (N_0_P_0_K_0_) were statistically insignificant. Unlike plant-available forms of TMs, the TEF for LCd and LPb was closer to 1 than for M3Cd and M3Pb (≈ 1.05). However, the TEF for LZn was exceptionally high compared to the others (≈ 3.12). A trend towards a relative increase in TEF for LZn and LCu due to the use of PS was observed. With regard to LCu, the effect of PS fertilization was unambiguous. With respect to Zn, in plots without PS, increasing doses of NPK decreased TEF. The opposite phenomenon was observed in PS plots. The smallest TEF for LZn was found in the PS + N_1_P_1_K_1_ treatment (Fig. 1).

### Metal content as a function of soil properties

A correlation analysis was performed within the variability caused only by fertiliser factors to discard the influence of the seasonal factor. The results of the analysis are shown in Table 8. Soil reaction (pH) was positively correlated with calcium (M3Ca) content. A significant negative correlation was found between pH and TN, regardless of soil depth. In topsoil, a significant negative dependence of M3P on pH was also found. Moreover, TSC negatively correlated with pH, although the correlation coefficient was not statistically significant. As regards TMs, the bioavailable forms correlated more strongly with basic soil parameters than pseudo-total content. The exception was TZn, which correlated positively with the TSC and TN, and negatively with M3Ca in the topsoil. In addition, the TPb correlated positively with the TN content in subsoil. With respect to plant-available forms of TMs, only M3Zn significantly correlated with the TN in the topsoil. Contrary to topsoil, in subsoil more significant correlation coefficients were obtained for metals determined by the Mehlich 3 method. The M3Zn content correlated negatively with pH and positively with TN, M3P and M3K. In turn, M3Cd correlated significantly and positively with TSC and TN.

**Table 8:** Correlation matrix—relationship between the mean content of trace metals and the basic agrochemical properties of the soil (*n* = 8)

Parameters	pH	TSC	TN	M3P	M3K	M3Ca	M3Mg
Topsoil (0.0–0.3 m)							
TSC	-0.68						
TN	-0.91^**^	0.89^**^					
M3P	-0.72^*^	0.88^**^	0.93^***^				
M3K	-0.63	0.66	0.77^*^	0.90^**^			
M3Ca	0.94^***^	-0.71^*^	-0.95^***^	-0.81^*^	-0.66		
M3Mg	0.17	0.19	-0.09	-0.24	-0.60	0.17	
TCd	0.14	0.14	0.03	0.18	0.23	0.10	-0.06
TCu	-0.01	-0.20	-0.10	-0.27	-0.44	0.08	0.39
TPb	0.59	-0.13	-0.53	-0.46	-0.48	0.70	0.51
TZn	-0.66	0.82^*^	0.80^*^	0.67	0.33	-0.72^*^	0.45
M3Cd	-0.59	0.50	0.53	0.32	0.17	-0.57	0.26
M3Cu	-0.11	0.64	0.41	0.41	0.02	-0.29	0.70
M3Pb	0.37	0.06	-0.09	0.08	-0.00	0.14	0.16
M3Zn	-0.38	0.82^*^	0.65	0.62	0.24	-0.52	0.57
LCd	-0.96^***^	0.78^*^	0.94^***^	0.79^*^	0.63	-0.92^**^	-0.04
LCu	-0.06	-0.17	-0.04	-0.22	-0.42	-0.06	0.36
LPb	-0.34	0.24	0.42	0.59	0.78^*^	-0.41	-0.74^*^
LZn	-0.88^**^	0.92^**^	0.96^***^	0.86^**^	0.66	-0.87^**^	0.07
Subsoil (0.3–0.6 m)							
TSC	-0.02						
TN	-0.78^*^	0.43					
M3P	-0.65	0.27	0.80^*^				
M3K	-0.70	0.10	0.77^*^	0.97^***^			
M3Ca	0.97^***^	-0.04	-0.70	-0.53	-0.58		
M3Mg	-0.06	-0.73^*^	-0.03	-0.09	0.08	0.05	
TCd	0.01	0.30	0.45	0.33	0.18	0.03	-0.14
TCu	-0.60	-0.19	0.63	0.59	0.66	-0.51	0.41
TPb	-0.64	0.01	0.79^*^	0.57	0.66	-0.55	0.46
TZn	-0.58	-0.30	0.61	0.32	0.39	-0.52	0.63
M3Cd	-0.29	0.75^*^	0.74^*^	0.62	0.43	-0.23	-0.45
M3Cu	-0.48	0.14	0.76^*^	0.64	0.68	-0.40	0.27
M3Pb	0.10	0.90^**^	0.43	0.35	0.20	0.12	-0.56
M3Zn	-0.73^*^	-0.06	0.78^*^	0.76^*^	0.84^**^	-0.64	0.35
LCd	-0.95^***^	0.11	0.81^*^	0.77^*^	0.84^**^	-0.90^**^	0.03
LCu	0.72^*^	0.40	-0.31	-0.41	-0.57	0.74^**^	-0.19
LPb	-0.09	0.73^*^	0.35	0.20	-0.01	-0.12	-0.67
LZn	-0.88^**^	0.13	0.80^*^	0.75^*^	0.81^*^	-0.78^*^	0.16

^***^, **, * significant at *p* ≤ 0.001; *p* ≤ 0.01; *p* ≤ 0.05, respectively. Key: TSC – total soil carbon, TN – total nitrogen, M3 – plant-available content (Mehlich 3) of elements, T – pseudo-total content (*Aqua regia*) of metals, L – mobile content (1 M NH_4_NO_3_) of metals

**Fig. 2:**
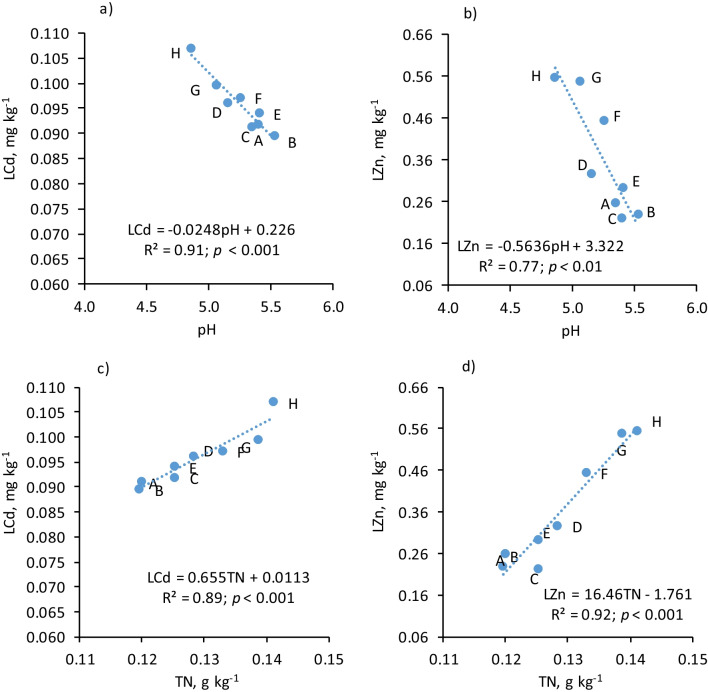
Dependence of the content of mobile Cd and Zn (LCd and LZn) on soil pH and TSC content in the topsoil. Treatments: A – control; B – N_1_P_1_K_1_; C – N_2_P_2_K_2_; D – N_4_P_2_K_2_; E – pig slurry (PS); F – PS + N_1_P_1_K_1_; C – PS + N_3_P_2_K_2_; D – PS + N_4_P_2_K_2_

In topsoil, mobile forms of Cd and Zn (LCd and LZn) were correlated with pH, TSC, TN, M3Ca as well as M3P. As the pH increased, the amount of mobile TM forms decreased (Fig. 2). The opposite relationship was observed for TSC, TN and M3P. Among them, the LCd and LZn content was mainly determined by the TN content. In subsoil, pH also correlated negatively with the content of LCd and LZn. In contrast to topsoil, in subsoil, the content of mobile Cd and Zn correlated with the content of TN to an even greater extent than with TSC (Table 8, Fig. 2).

## Discussion

The permissible levels of Cd, Cu, Pb and Zn in clay-textured mineral soils in the Czechia are: 0.5, 60, 60 and 120 mg kg^−1^ (Vácha et al. 2014). Thus, as proven in our studies, more than 65–66 years of using PS and different doses of mineral fertilizers (NPK) did not exceed the standards for the total TM content in the soil. The greatest risk of exceeding the above standards was noted for Cd, in particular in topsoil. Previous soil tests from the analysed RFE long-term experiment did not show that the permissible standards for such elements as Cu, Pb and Zn were exceeded (Uprety et al. 2009). On the other hand, total Cd concentrations in soil exceeded the Czech legislative limit. According to the authors, this was also the case for the soil samples collected from the control treatment – without fertilizers, which clearly indicated that the main source of Cd in the soil was the atmosphere, not fertilizers. Zhao et al. (2018) obtained similar evidence in a long-term experiment conducted in China. The lower Cd content in the soil samples analysed in our study indicates a possible reduction in the supply of Cd from the atmosphere, as well as the removal of this metal from topsoil in the crop yield and/or its movement to deeper soil layers. According to Hůnová et al. (2023), the total atmospheric deposition of Cd in the Czechia decreased significantly in the period 1996–2021 as a result of the reduction of fossil fuel combustion and dust emissions. The highest Cd deposition of 21 t over the whole territory of the Czechia was recorded in 1996; the lowest deposition of 2.6 t was observed in 2018. In terms of spatial distribution, Cd deposition from the atmosphere decreased significantly in the southern parts of the Czechia, including the Prague area.

Long-term mineral-organic fertilization did not have a significant impact on the pseudo-total Cu and Zn content. However, a trend towards an increase in the TZn content was observed in soil samples cyclically fertilized with PS, regardless of the soil depth. According to the literature, long-term use of slurry increases the content of these metals in the soil (Mantovi et al. 2003; Leclerc and Laurent 2017). According to Kumaragamage et al. (2016) the significant impact of organic fertilization on the content of Zn and Cu results from the fact that the introduced loads of both elements are usually above crop removal rates and, as a consequence, their systematic accumulation occurs. There is data in the literature indicating that after the first year of the application of slurry, differences in the content of Cu and Zn may appear in relation to the control. According to Lan et al. (2022) after 4 years of the experiment, the differences from the controls were about 67% and 39%, respectively for Cu and Zn. Shakoor et al. (2022) showed that the annual application of PS at doses of 20, 40 and 80 m^3^ (146–534 kg N ha^−1^) for 7 years significantly increased the content of Cu and Zn in topsoil, but had no effect on the content of Pb. According to Tiecher et al. (2013) annual application of pig manure can lead not only to an increase in the content of Cu and Zn in topsoil, but also in subsoil, especially when the dose of this fertilizer is relatively high. On the other hand, in a long-term experiment (36–37 years) in Poland, it was shown that the degree of the impact of cattle slurry fertilization on the content of TMs depends on the type of soil (Mazur and Mazur 2016). This factor significantly increased the content of total Cu in lessive soil, and Zn in brown soil. As per the cited authors, the use of varying NPK rates had less effect on the concentration of TCu and TZn than slurry fertilization. This effect of fertilizers was also confirmed by the present study.

In the experiment carried out, fertilization with slurry significantly influenced the content of TZn, not TCu. The main reason for the difference was the amount of input of both TMs into the soil. According to Nicholson et al. (2003) together with a slurry application about 2321 g ha^−1^ Zn and 1679 g ha^−1^ Cu can be introduced into the soil per year. Other authors suggest even higher annual loads of Zn and Cu flowing into the soil as a result of the application of PS, e.g. 15,000 and 4000 g, respectively for Zn and Cu (Moral et al. 2008). In our own research, the inflow of Zn in the year of PS application was comparable to the data of Nicholson et al. (2003), but Cu was lower, which was due to the lower concentration of the element in the PS. In results, the average annual input of both elements in the one crop rotation amounted to 550 g ha^−1^ Zn and 156 g ha^−1^ Cu. The lack of a significant effect of PS application on TCu content may be due to the four times lower influx of Cu to the soil than Zn and the balance of influx with metal removal in plant yield. Some authors emphasize greater problems with the determination of TCu than TZn using *aqua regia*, but these generally apply to soils with very high organic matter content (McGrath et al. 1988).

In the studies, no significant effect of fertilization on the pseudo-total content of Cd and Pb was observed. The lack of significant differences in the content of TMs as a result of many years of mineral fertilization was also observed by other authors (Uprety et al. 2009; Ajayi et al. 2012). However, there is data in the literature indicating that long-term use of mineral fertilizers significantly increases the total content of TMs in the soil, including Cd (Thomas et al. 2012; Czarnecki and Düring 2015; Park et al. 2021). One of the reasons for the differences in the observed results may be the type of soil (Mazur and Mazur 2016). Among mineral fertilizers, fertilizers containing phosphorus (P) have relatively high amounts of Cd (Suciu et al. 2022). According to some authors, as P doses increase, the concentration of total Cd in the soil increases (Wang et al. 2014; Czarnecki and Düring 2015;). Studies carried out by Mar et al. (2012) showed that increasing doses of superphosphate (up to 120 kg ha^−1^) did not significantly differentiate the content of total Cd (as well as other forms of Cd) compared to the controls. However, the authors observed a trend towards an increase in the content of this nutrient in the soil, especially in its water-soluble form. Long-term studies (> 50 years) indicate that if phosphate rock contains less than 10 mg kg^−1^ Cd, the risk of soil and plant contamination with this element is negligible (Mortvedt 1995). In our study, the total Cd content in superphosphate was about 7.82 mg kg^−1^. As previous studies indicate, low Cd concentration in phosphorus fertilizers does not result in a significant increase in the concentration of this element in the soil, even after 30 years of systematic use of P in doses of 50, 100 and 150 kg P_2_O_5_ ha^−1^ (Bogdanovic et al. 1999). According to Dharma-Wardana (2018), taking into account standard NPK fertilizers and agricultural practices, it will take hundreds of years to double the total Cd content of the soil. Compared to P fertilizers, PS contains little Cd and does not pose a major threat to soil quality (Nicholson et al. 2003). However, some data found in the literature indicate that as early as the second year of application of pig slurry a significant increase in the content of Cd (and Pb) in the soil is possible compared to the control Lan et al. (2022). The lack of a significant effect of NPK and PS fertilization on the content of Cd in the soil could result from balancing the inputs and outputs of this element. The leaching of Cd from the soil may amount to 1.1–1.8 g ha^−1^ (McDowell 2022). The removal rates of Cd through crop uptake may range from 0.15 to 4.4 g ha^−1^ year^−1^ (Čásová et al. 2009; Xu et al. 2015). Thus, in our own studies, the potential removal with plant yield and leaching could be greater than the average annual load of Cd introduced into the soil in fertilizers (maximum 2.06 g ha^−1^ year^−1^).

The content of bioavailable forms of TMs was analysed using the Mehlich 3 method and by using 1 M NH_4_NO_3_ solution. The first method is used in many countries for routine determination of soil micronutrients, including, of course, Zn and Cu (Zbíral and Němec 2000). The use of this method is supported by the fact that Mehlich 3 solution demonstrated a greater capacity of extraction of Zn and Cu in comparison to the other extractants (Pradhan et al. 2015). Therefore, the above method can also be used to assess the degree of soil contamination with these elements (Zhang et al. 2010). For Czech soils, the critical content of MCd and MCu was determined to be 0.27 mg kg and 13 mg kg, respectively (Malý et al. 2021). Therefore, the content of both elements analyzed by the Mehlich3 method in our study did not exceed the proposed limit.

In our study, in contrast to pseudo-total forms of TMs, the content of plant-available forms of Cu, Pb and Zn in the topsoil depended significantly on the growing season. These differences can be explained by the direct use of PS (in the 2020/2021) and/or soil factors increasing the pool of mobile forms of elements. Among the analysed elements, only the M3Zn content significantly depended on the fertilizer factor. Regardless of mineral NPK treatments, the application of PS increased the average M3Zn concentration by 55.7% in the topsoil and 26.9% in the subsoil. The application of the PS also resulted in an increase in the M3Cu content. The differences were not statistically significant compared to the plots without PS. This result can be explained by a lower influx of metal into the soil as well as the formation of more stable organo-mineral complexes by Cu than by Zn, which makes the determination of bioavailable copper difficult (Bradl 2004). In contrast to Zn and Cu, the contents of Cd and Pb were only slightly affected by PS fertilization. Compared to the effects of PS, the effect of NPK doses was very small. In comparison, Pradhan et al. (2015) showed, by analysing 12 different long-term experiments in India, that fertilisation with NPK mineral fertilizers increases the soil M3Zn and M3Cu concentration compared to the control. However, a treatment with mineral and organic fertilizers had the greatest impact on their content.

Limit values for heavy metals determined by the 1 M NH_4_NO_3_ method were developed in the Czechia for As, Cd, Ni, Pb, Tl and Hg. They depend on pH and soil texture. For the soil tested in the experiment they are 0.1 mg kg^−1^ for Cd and 1.5 mg kg^−1^ for Pb (Vácha et al. 2014). Therefore, the LCd levels obtained in the studies oscillated around the critical level that protects against potential contamination of the food chain. The recommended limit was even slightly exceeded in the topsoil in treatment PS + N_4_P_2_K_2_. In a similar way to the plant-available, the content of mobile forms of TMs in topsoil depended on the growing season. Higher values were obtained in 2021. Of all the analysed metals, only the content of LZn was significantly shaped by the fertilizer factor. On average, the highest concentration of LZn was found in the PS + N_4_P_2_K_2_ treatment. Moreover, there was a tendency to increase the content of LPb and LCd in treatments with higher NPK doses. Our results are consistent with the previous studies of Uprety et al. (2009). The authors showed that long-term mineral-organic fertilization significantly affected only mobile Zn. However, the authors did not prove a significant impact of fertilization on the content of other TMs in mobile form. Our results are also confirmed by other long-term field studies (Lehoczky et al. 2004). Czarnecki and Düring (2015), in turn, found that a 14-year application of NPK fertilizers considerably increases the mobile form content of Cd, Cu, Pb and Zn, in comparison to the control.

In our study, the percentage of bioavailable TMs in their total content depended on the tested element, analytical method and soil depth. The largest portion of this form in the total content was found for LCd, regardless of whether the element was determined using the Mehlich 3 method or using the NH_4_NO_3_ solution. This is in line with earlier results obtained by Czarnecki and Düring (2015) and Malý et al. (2021). One of the reasons for such a relationship is the weaker strength of Cd binding by the soil sorption complex compared to other elements, especially in acidic pH (Gu et al. 2022). Generally, Zn is also a mobile element in the soil. However, the proportion of its bioavailable forms in the pseudo-total content was much lower than for Cd. Despite this, only the share of bioavailable Zn changed significantly under the influence of fertilization. This was due to two facts: a large inflow of Zn together with PS and the formation of soil conditions favouring Zn mobility. Regarding the last comment, it is worth emphasizing that the highest proportion of the LZn in TZn was obtained as a result of the simultaneous application of PS and the highest dose of NPK. Unlike Cd and Zn, Pb and Cu are strongly bound to SOM. This reduces the risk of transforming their total to bioavailable forms (Campillo-Cora et al. 2020; Deng et al. 2023). However, compared to Cu, Pb has greater bioavailability potential and consequently a greater risk of contamination and entry into the food chain (Coutinho et al. 2020). Our studies indirectly confirm this, as the proportions of bioavailable forms of Pb in the pseudo-total were higher than for Cu.

According to numerous studies, the soil texture, pH, Eh, CaCO_3_ and SOC are the main parameters determining the content of heavy metals and metalloids in different fractions and forms (Sungur et al. 2014; Popenda 2014; Zhang et al. 2020; Gu et al. 2022). These factors generally affect soluble and bioavailable forms. The content of total forms depends directly on the input of TMs from anthropogenic sources. This is confirmed by correlation analysis. Only the TZn content positively correlated with TSC, because the PS was a source of both organic carbon and this element in a relatively large dose. In the conducted research, TSC should be considered mainly as SOC, because the CaCO_3_ content in the soil was trace. The SOC is the main source of active sites that have strong complexation ability with TMs (Li et al. 2023). Moreover, at the same time, the organic compounds that are the source of SOC undergo mineralization and are a source of H^+^ protons (Neina 2019). As a result, a negative relationship between soil pH and SOC can be observed (Czarnecki and Düring 2015; Zhou et al. 2019). According to Zhang et al. (2010) pH had more significant influence on the content of bioavailable forms of TMs in the soils than other soil properties, including SOC. As a consequence, the more acid the soil, the higher amount of soluble and bioavailable forms of TMs (Tkaczyk et al. 2017; Al-Qasi et al. 2021). Our own research also confirms this phenomenon, especially in relation to TMs determined using the NH_4_NO_3_ solution. The LCd and LZn significantly and negatively correlated with pH. The opposite relationship was observed for TSC. In addition, the TN parameter better correlated with mobile TMs than TSC, especially in subsoil. PS was a source not only of carbon organic compounds undergoing mineralization and humification, but also organic and mineral forms of N, including NH_4_^+^ ions. As a result of N organic decomposition, nitrification of NH_4_^+^ ions, and/or its direct uptake by plants, the soil pH decreased. Mineral fertilizers containing NH_4_^+^ ions also decrease the soil pH. In the experiment, calcium ammonium nitrate had been used for many years. Compared to urea or ammonium nitrate, it can be considered as near-neutral in its effect on soil pH (Kissel et al. 2020). However, it was systematically added to the soil. In addition, N fertilization increases the biomass of crop residues and thus the inflow of organic matter to the soil (Mazzoncini et al. 2011). Field tests conducted in Poland clearly show that 96 years of using NPK mineral fertilizers leads to a decrease in soil pH (Viet 2023). The decrease in pH resulted in an increase in the amount of Cd and Zn forms poorly bound to the soil. This phenomenon has not been confirmed for Cu and Pb. The weak relationships between extractable forms of Cu, Pb and soil pH have also been reported in previous studies (Kupka et al. 2021). The results could be attributed to the strong affinity for soil organic matter by these metals (Brümmer and Herms 1983). The positive relationship between the mobile forms of Cd and Zn and the content of M3P can be explained by the fact that P fertilizers are carriers of both elements. However, the positive correlation of the content of LCd and LZn with TN suggests an indirect mechanism, through the positive effect of the use of N and P fertilizers on the input of organic matter to soil.

Long-term use of PS and NPK mineral fertilizers did not have a significant impact on the topsoil enrichment factor for pseudo-total forms of metals. With regard to bioavailable forms, in plots with PS an increase in the average concentration of Zn and Cu in topsoil compared to subsoil was found, especially in forms extracted with NH_4_NO_3_ solution. At the same time, increasing doses of NPK decreased the TEF for LZn in the facility without slurry, which may indicate the following phenomena: greater demand of plants for Zn in conditions of using high doses of NPK, formation of insoluble phosphates and/or movement of soluble forms towards the subsoil. The lack of such a relationship in the plots with PS and NPK fertilizers proves that the first fertilizer both provides plants with this microelement and creates optimal conditions for its accumulation by increasing the content of SOC. Regarding the mobile forms of TMs, in plots without PS, increasing doses of NPK decreased the TEF for LZn. This may be due to the increase in plant biomass and the metal uptake by the plant. Another reason could be the formation of poorly mobile Zn phosphates. The opposite phenomenon was observed in PS plots. The lowest TEF for LZn was found in the PS + N_0_P_0_K_0_ treatment. This result clearly indicates that only the simultaneous application of PS and NPK fertilizers promotes the accumulation in the topsoil of Zn forms readily available to plants, in amounts sufficient to cover the plants' needs under conditions of increasing NPK doses.

## Conclusion

Long-term (65 years) annual fertilization with mineral fertilizers (NPK) and regular application of slurry for root crops in a 9-year crop rotation does not pose a threat to either soil quality or heighten the risk of excessive amounts of trace metals (Cd, Cu, Pb and Zn) entering the food chain. The pseudo-total TMs content in the topsoil and subsoil was below the permissible critical values required in the Czechia. The long-term fertilization significantly changed only the Zn content in the soil, especially the bioavailable forms. The dominant effect on the content of this metal was exerted by the application of pig slurry. In the plots with pig slurry, the mobile Zn content also increased with increasing NPK fertilizer doses. Despite this, the increase in mobile Zn does not represent a major threat to soil quality, as it is an essential micronutrient for plants and consumers. However, a worrying result could be the tendency to increase the content of readily bioavailable (mobile) forms of Pb and Cd due to the use of NPK fertilizers. Under the conditions of the experiment, the risk of contamination of the food chain is of particular concern for Cd. This is due to two facts: the average content of its mobile (soluble in 1 M NH_4_NO_3_) form in the soil oscillated around the recommended critical content, and the long-term application of PS and NPK fertilizers decreased the soil pH, thereby increasing the bioavailability of the metal.

## Supplementary Information

Below is the link to the electronic supplementary material.

Supplementary file1 (DOCX 34.6 KB)

## Data Availability

All data generated or analyzed during this study are included in this published article [and its supplementary information files].
