# Supplementary material for: Effect of long-term application of pig slurry and NPK fertilizers on trace metal content in the soil
Source: Environ Sci Pollut Res Int. 2024 Oct 4;31(50):60004–22. doi: 10.1007/s11356-024-34993-1 (PMC11519191; doi:10.1007/s11356-024-34993-1)
Supplement: Supplementary file 1 — Supplementary file1 (DOCX 34.6 KB) [file 11356_2024_34993_MOESM1_ESM.docx]

**Supplementary material (information)**

**Effect of long-term application of pig slurry and NPK fertilizers on trace metal content in the soil**

**Przemysław Barłóg^1^*, Lukáš Hlisnikovský^2^ _,_ Remigiusz Łukowiak^1^ and Eva Kunzová^2^**

^1^Department of Agricultural Chemistry and Environmental Biogeochemistry, Poznań University of Life Sciences, Wojska Polskiego 71F, 60-625 Poznań, Poland

^2^Department of Nutrition Management, Crop Research Institute, Drnovská 507, CZ˗161 01 Prague 6, Ruzyně, the Czech Republic

***** Correspondence: przemyslaw.barlog@up.poznan.pl

**Table S1** Mean annual rates of the main macronutrients (NPK) in mineral fertilizers and pig slurry over one nine years crop rotation (kg ha^-1^)

| Treatments | Alfalfa | Alfalfa | Winter wheat | Sugar beet | Spring barley | Potatoes | Winter wheat | Sugar beet | Spring barley | Mean |
| --- | --- | --- | --- | --- | --- | --- | --- | --- | --- | --- |
| N_0_P_0_K_0_ | 0-0-0 | 0-0-0 | 0-0-0 | 0-0-0 | 0-0-0 | 0-0-0 | 0-0-0 | 0-0-0 | 0-0-0 | 0-0-0 |
| N_1_P_1_K_1_ | 0-22-133 | 0-31-183 | 40-21-80 | 80-28-125 | 30-21-66 | 50-22-120 | 40-21-80 | 80-28-125 | 30-21-66 | 39-24-109 |
| N_3_P_2_K_2_ | 0-31-183 | 0-43-249 | 55-26-100 | 160-35-166 | 50-26-83 | 70-31-186 | 55-26-100 | 160-35-166 | 50-26-83 | 67-31-146 |
| N_4_P_2_K_2_ | 0-31-183 | 0-43-249 | 75-26-100 | 200-35-166 | 70-26-83 | 110-31-186 | 75-26-100 | 200-35-166 | 70-26-83 | 91-31-146 |
| PS+N_0_P_0_K_0_ | 0-0-0 | 0-0-0 | 0-0-0 | 89-39-88 | 0-0-0 | 64-28-63 | 0-0-0 | 89-39-88 | 0-0-0 | 27-12-27 |
| PS+N_1_P_1_K_1_ | 0-22-133 | 0-31-183 | 40-21-80 | 169-67-213 | 30-21-66 | 114-50-183 | 40-21-80 | 169-67-213 | 30-21-66 | 66-36-135 |
| PS+N_3_P_2_K_2_ | 0-31-183 | 0-43-249 | 55-26-100 | 249-74-254 | 50-26-83 | 134-59-249 | 55-26-100 | 249-74-254 | 50-26-83 | 94-43-173 |
| PS+N_4_P_2_K_2_ | 0-31-183 | 0-43-249 | 75-26-100 | 289-74-254 | 70-26-83 | 174-59-249 | 75-26-100 | 289-74-254 | 70-26-83 | 116-43-173 |

PS – pig slurry at the rate 68 t ha^-1^ (sugar beet) and 49 t ha^-1^ (potatoes).

**Table S2** Two-way ANOVA results – effect of long-term application of pig slurry and NPK fertilizers on trace metals (TMs) content in topsoil and subsoil

| Factor | Degrees of freedom | Pseodo-total  (*Aqua regia*) | | | | Plant-available  (Mehlich 3) | | | | Mobile  (NH_4_NO_3_) | | | |
| --- | --- | --- | --- | --- | --- | --- | --- | --- | --- | --- | --- | --- | --- |
|  |  | Cu | Zn | Pb | Cd | Cu | Zn | Pb | Cd | Cu | Zn | Pb | Cd |
| Topsoil: 0.0-0.3 m | | | | | | | | | | | | | |
| Year (Y) | 1 | n.s. | n.s. | n.s. | n.s. | *** | *** | *** | n.s. | n.s. | *** | *** | * |
| Treatm.(T) | 7 | n.s. | n.s. | n.s. | n.s. | n.s. | *** | n.s. | n.s. | n.s. | *** | n.s. | n.s. |
| Y x T | 7 | n.s. | n.s. | n.s. | n.s. | n.s. | n.s. | n.s. | n.s. | n.s. | n.s. | n.s. | n.s. |
| Error | 48 |  |  |  |  |  |  |  |  |  |  |  |  |
| Subsoil: 0.3-0.6 m | | | | | | | | | | | | | |
| Year (Y) | 1 | *** | n.s. | *** | * | *** | *** | *** | n.s. | n.s. | *** | *** | ** |
| Treatm.(T) | 7 | n.s. | n.s. | n.s. | n.s. | n.s. | n.s. | n.s. | n.s. | n.s. | n.s. | n.s. | n.s. |
| Y x T | 7 | n.s. | n.s. | n.s. | n.s. | n.s. | n.s. | n.s. | n.s. | n.s. | n.s. | n.s. | n.s. |
| Error | 48 |  |  |  |  |  |  |  |  |  |  |  |  |

***, **, * significant at *p* < 0.001; *p* < 0.01; *p* < 0.05, respectively; n.s. – non significant

**Table S3**. Effect of fertilization treatments on the pseudo-total (*Aqua regia*) trace metals concentrations in soil samples (mean ± SEM) depending on the year and soil depth (mg kg^-1^)

| Treatments | 2020 | | | | 2021 | | | |
| --- | --- | --- | --- | --- | --- | --- | --- | --- |
|  | Cd | Cu | Pb | Zn | Cd | Cu | Pb | Zn |
| Topsoil: 0.0-0.3 m | | | | | | | | |
| Control | 0.36 ± 0.07 | 30.3 ± 1.8 | 26.1 ± 5.6 | 61.5 ± 3.4 | 0.24 ± 0.08 | 27.6 ± 2.9 | 20.2 ± 1.7 | 64.6 ± 0.8 ^ab^ |
| N_1_P_1_K_1_ | 0.35 ± 0.07 | 29.3 ± 2.0 | 26.1 ± 2.4 | 58.2 ± 4.3 | 0.46 ± 0.10 | 28.0 ± 0.7 | 21.1 ± 2.3 | 66.1 ± 1.1 ^ab^ |
| N_3_P_2_K_2_ | 0.38 ± 0.06 | 28.6 ± 1.8 | 19.1 ± 4.7 | 62.0 ± 3.6 | 0.48 ± 0.18 | 27.3 ± 2.1 | 23.7 ± 3.0 | 65.6 ± 1.8 ^ab^ |
| N_4_P_2_K_2_ | 0.35 ± 0.07 | 30.5 ± 2.9 | 20.7 ± 4.3 | 67.1 ± 7.0 | 0.30 ± 0.11 | 27.2 ± 0.4 | 20.9 ± 2.4 | 61.4 ± 2.3 ^b^ |
| PS | 0.44 ± 0.07 | 29.4 ± 1.7 | 23.2 ± 2.5 | 69.4 ± 8.0 | 0.40 ± 0.24 | 29.5 ± 1.6 | 21.4 ± 2.4 | 71.0 ± 3.0 ^a^ |
| PS+N_1_P_1_K_1_ | 0.30 ± 0.02 | 30.6 ± 1.9 | 23.7 ± 2.2 | 65.1 ± 2.6 | 0.35 ± 0.12 | 28.3 ± 1.8 | 20.5 ± 2.0 | 71.2 ± 2.4 ^a^ |
| PS+N_3_P_2_K_2_ | 0.32 ± 0.02 | 26.1 ± 1.1 | 22.8 ± 3.8 | 67.1 ± 4.6 | 0.40 ± 0.07 | 28.1 ± 1.4 | 21.9 ± 1.3 | 71.6 ± 2.2 ^a^ |
| PS+N_4_P_2_K_2_ | 0.35 ± 0.05 | 30.1 ± 1.7 | 20.9 ± 1.3 | 71.1 ± 9.0 | 0.47 ± 0.10 | 28.9 ± 1.0 | 21.7 ± 1.6 | 71.7 ± 0.7 ^a^ |
| Subsoil: 0.3-0.6 m | | | | | | | | |
| Control | 0.38 ± 0.08 | 31.3 ± 0.6 | 23.5 ± 4.7 | 63.4 ± 3.5 | 0.15 ± 0.06 | 25.4 ± 0.4 | 15.1 ± 1.6 | 64.9 ± 2.5 |
| N_1_P_1_K_1_ | 0.38 ± 0.08 | 29.4 ± 0.9 | 24.1 ± 5.6 | 61.2 ± 1.4 | 0.28 ± 0.08 | 25.3 ± 1.3 | 12.9 ± 2.8 | 64.7 ± 0.3 |
| N_3_P_2_K_2_ | 0.37 ± 0.08 | 30.7 ± 0.9 | 22.5 ± 6.7 | 62.9 ± 1.1 | 0.31 ± 0.08 | 24.9 ± 1.6 | 12.7 ± 2.4 | 64.0 ± 3.7 |
| N_4_P_2_K_2_ | 0.40 ± 0.05 | 30.8 ± 1.2 | 24.3 ± 4.6 | 60.9 ± 2.5 | 0.20 ± 0.10 | 25.1 ± 0.7 | 14.9 ± 1.5 | 62.9 ± 1.0 |
| PS | 0.34 ± 0.04 | 30.1 ± 1.8 | 25.6 ± 1.3 | 74.2 ± 9.5 | 0.31 ± 0.11 | 26.1 ± 1.3 | 15.3 ± 0.9 | 66.5 ± 1.5 |
| PS+N_1_P_1_K_1_ | 0.31 ± 0.04 | 34.1 ± 0.4 | 27.6 ± 0.7 | 71.0 ± 3.6 | 0.22 ± 0.06 | 24.3 ± 0.9 | 12.2 ± 1.0 | 57.7 ± 6.2 |
| PS+N_3_P_2_K_2_ | 0.35 ± 0.02 | 31.8 ± 0.9 | 26.2 ± 2.0 | 66.6 ± 2.5 | 0.22 ± 0.17 | 25.6 ± 1.4 | 14.0 ± 2.2 | 65.9 ± 2.7 |
| PS+N_4_P_2_K_2_ | 0.37 ± 0.07 | 34.0 ± 0.5 | 30.9 ± 3.2 | 71.4 ± 4.6 | 0.35 ± 0.22 | 27.2 ± 1.3 | 14.2 ± 3.1 | 68.2 ± 0.5 |

Different letters indicate statistically significant differences between treatments at *p* < 0.05 (HSD test; one-way ANOVA).

**Table S4**. Effect of fertilization treatments on the plant-available (Mehlich 3) trace metals concentrations in soil samples (mean ± SEM) depending on the year and soil depth (mg kg^-1^)

| Treatments | 2020 | | | | 2021 | | | |
| --- | --- | --- | --- | --- | --- | --- | --- | --- |
|  | Cd | Cu | Pb | Zn | Cd | Cu | Pb | Zn |
| Topsoil: 0.0-0.3 m | | | | | | | | |
| Control | 0.18 ± 0.02 | 3.94 ± 0.79 | 5.29 ± 1.19 | 2.47 ± 0.63 | 0.19 ± 0.01 | 5.69 ± 0.63 | 7.37 ± 0.25 | 4.88 ± 0.26 ^bcd^ |
| N_1_P_1_K_1_ | 0.17 ± 0.02 | 4.22 ± 0.60 | 6.53 ± 0.71 | 2.80 ± 0.44 | 0.18 ± 0.02 | 5.34 ± 0.46 | 6.95 ± 0.87 | 4.63 ± 0.72 ^d^ |
| N_3_P_2_K_2_ | 0.18 ± 0.03 | 4.38 ± 0.64 | 6.72 ± 1.08 | 3.05 ± 0.47 | 0.18 ± 0.02 | 5.63 ± 0.66 | 8.74 ± 1.32 | 4.84 ± 0.25 ^cd^ |
| N_4_P_2_K_2_ | 0.17 ± 0.03 | 3.82 ± 1.01 | 5.30 ± 1.40 | 3.02 ± 0.99 | 0.18 ± 0.03 | 5.14 ± 0.84 | 6.89 ± 1.06 | 4.31 ± 0.53 ^d^ |
| PS | 0.19 ± 0.01 | 5.27 ± 0.25 | 6.99 ± 0.26 | 4.75 ± 0.34 | 0.18 ± 0.01 | 6.75 ± 0.21 | 8.01 ± 0.21 | 7.32 ± 0.58 ^ab^ |
| PS+N_1_P_1_K_1_ | 0.18 ± 0.01 | 4.95 ± 0.69 | 6.11 ± 0.82 | 4.63 ± 0.91 | 0.18 ± 0.01 | 6.51 ± 0.38 | 7.44 ± 0.17 | 7.30 ± 0.53 ^abc^ |
| PS+N_3_P_2_K_2_ | 0.19 ± 0.02 | 4.77 ± 0.84 | 5.84 ± 0.94 | 4.81 ± 1.10 | 0.18 ± 0.01 | 6.46 ± 0.55 | 7.73 ± 0.23 | 7.38 ± 0.63 ^a^ |
| PS+N_4_P_2_K_2_ | 0.19 ± 0.01 | 4.64 ± 0.75 | 6.34 ± 1.00 | 4.43 ± 1.22 | 0.20 ± 0.01 | 5.89 ± 0.87 | 6.97 ± 0.20 | 6.08 ± 0.54^abcd^ |
| Subsoil: 0.3-0.6 m | | | | | | | | |
| Control | 0.15 ± 0.01 | 2.72 ± 0.32 | 3.05 ± 0.50 | 1.35 ± 0.23 | 0.15 ± 0.02 | 4.32 ± 0.51 | 5.38 ± 0.42 | 3.74 ± 0.36 |
| N_1_P_1_K_1_ | 0.15 ± 0.01 | 3.49 ± 0.83 | 4.44 ± 0.69 | 2.20 ± 0.44 | 0.16 ± 0.02 | 4.70 ± 0.37 | 5.55 ± 0.54 | 3.87 ± 0.36 |
| N_3_P_2_K_2_ | 0.16 ± 0.01 | 3.03 ± 0.39 | 4.31 ± 0.79 | 1.85 ± 0.41 | 0.16 ± 0.02 | 4.27 ± 0.57 | 4.72 ± 0.54 | 3.57 ± 0.47 |
| N_4_P_2_K_2_ | 0.16 ± 0.01 | 3.13 ± 0.48 | 4.51 ± 0.90 | 2.09 ± 0.54 | 0.16 ± 0.02 | 4.88 ± 0.39 | 6.24 ± 0.44 | 4.10 ± 0.23 |
| PS | 0.16 ± 0.01 | 3.09 ± 0.25 | 3.69 ± 0.37 | 1.98 ± 0.17 | 0.15 ± 0.02 | 5.65 ± 0.56 | 5.46 ± 0.40 | 5.31 ± 0.64 |
| PS+N_1_P_1_K_1_ | 0.16 ± 0.01 | 3.27 ± 0.27 | 3.98 ± 0.51 | 2.49 ± 0.41 | 0.14 ± 0.02 | 4.53 ± 0.27 | 4.06 ± 0.39 | 4.15 ± 0.32 |
| PS+N_3_P_2_K_2_ | 0.16 ± 0.01 | 3.77 ± 0.28 | 4.32 ± 0.68 | 2.77 ± 0.48 | 0.15 ± 0.02 | 4.52 ± 0.46 | 4.89 ± 0.58 | 4.61 ± 0.64 |
| PS+N_4_P_2_K_2_ | 0.16 ± 0.01 | 3.97 ± 0.21 | 4.22 ± 0.78 | 2.63 ± 0.64 | 0.17 ± 0.02 | 5.00 ± 0.57 | 5.44 ± 0.75 | 4.93 ± 0.62 |

Different letters indicate statistically significant differences between treatments at *p* < 0.05 (HSD test; one-way ANOVA).

**Table S5**. Effect of fertilization treatments on the mobile (1 M NH_4_NO_3_) trace metals concentrations in soil samples (mean ± SEM) depending on the year and soil depth (mg kg^-1^)

| Treatments | 2020 | | | | 2021 | | | |
| --- | --- | --- | --- | --- | --- | --- | --- | --- |
|  | Cd | Cu | Pb | Zn | Cd | Cu | Pb | Zn |
| Topsoil: 0.0-0.3 m | | | | | | | | |
| Control | 0.078 ± 0.020 | 0.11 ± 0.06 | 0.17 ± 0.04 | 0.19 ± 0.03 | 0.104 ± 0.004 | 0.06 ± 0.03 | 0.18 ± 0.06 | 0.33±0.04 ^b^ |
| N_1_P_1_K_1_ | 0.080 ± 0.016 | 0.08 ± 0.01 | 0.17 ± 0.08 | 0.15 ± 0.04 | 0.099 ± 0.009 | 0.06 ± 0.02 | 0.31 ± 0.02 | 0.31±0.08 ^b^ |
| N_3_P_2_K_2_ | 0.083 ± 0.015 | 0.06 ± 0.03 | 0.24 ± 0.05 | 0.18 ± 0.04 | 0.101 ± 0.007 | 0.08 ± 0.01 | 0.31 ± 0.04 | 0.27±0.08 ^b^ |
| N_4_P_2_K_2_ | 0.087 ± 0.015 | 0.09 ± 0.02 | 0.23 ± 0.08 | 0.25 ± 0.05 | 0.105 ± 0.008 | 0.06 ± 0.02 | 0.31 ± 0.02 | 0.40±0.12 ^ab^ |
| PS | 0.086 ± 0.013 | 0.08 ± 0.01 | 0.12 ± 0.04 | 0.16 ± 0.06 | 0.102 ± 0.011 | 0.07 ± 0.01 | 0.33 ± 0.06 | 0.43±0.04 ^ab^ |
| PS+N_1_P_1_K_1_ | 0.097 ± 0.009 | 0.06 ± 0.02 | 0.11 ± 0.06 | 0.31 ± 0.10 | 0.097 ± 0.013 | 0.12 ± 0.01 | 0.33 ± 0.05 | 0.60±0.10 ^ab^ |
| PS+N_3_P_2_K_2_ | 0.095 ± 0.009 | 0.06 ± 0.01 | 0.21 ± 0.02 | 0.41 ± 0.08 | 0.104 ± 0.008 | 0.07 ± 0.01 | 0.30 ± 0.03 | 0.68±0.11 ^ab^ |
| PS+N_4_P_2_K_2_ | 0.105 ± 0.010 | 0.07 ± 0.00 | 0.23 ± 0.03 | 0.32 ± 0.13 | 0.110 ± 0.007 | 0.08 ± 0.02 | 0.29 ± 0.04 | 0.79±0.10 ^a^ |
| Subsoil: 0.3-0.6 m | | | | | | | | |
| Control | 0.074 ± 0.018 | 0.09 ± 0.02 | 0.18 ± 0.07 | 0.03 ± 0.01 | 0.095 ± 0.007 | 0.07±0.02 | 0.28 ± 0.02 | 0.12 ± 0.03 |
| N_1_P_1_K_1_ | 0.070 ± 0.014 | 0.07 ± 0.02 | 0.14 ± 0.08 | 0.03 ± 0.01 | 0.095 ± 0.011 | 0.07±0.02 | 0.28 ± 0.08 | 0.11 ± 0.05 |
| N_3_P_2_K_2_ | 0.080 ± 0.014 | 0.05 ± 0.01 | 0.20 ± 0.07 | 0.04 ± 0.01 | 0.093 ± 0.004 | 0.09±0.02 | 0.31 ± 0.03 | 0.11 ± 0.05 |
| N_4_P_2_K_2_ | 0.082 ± 0.015 | 0.07 ± 0.01 | 0.22 ± 0.05 | 0.05 ± 0.01 | 0.108 ± 0.010 | 0.06±0.01 | 0.31 ± 0.06 | 0.21 ± 0.07 |
| PS | 0.086 ± 0.009 | 0.03 ± 0.02 | 0.10 ± 0.07 | 0.05 ± 0.01 | 0.102 ± 0.011 | 0.06±0.01 | 0.31 ± 0.02 | 0.24 ± 0.05 |
| PS+N_1_P_1_K_1_ | 0.088 ± 0.010 | 0.04 ± 0.02 | 0.12 ± 0.06 | 0.06 ± 0.01 | 0.103 ± 0.010 | 0.03±0.02 | 0.29 ± 0.03 | 0.18 ± 0.05 |
| PS+N_3_P_2_K_2_ | 0.091 ± 0.013 | 0.05 ± 0.02 | 0.19 ± 0.04 | 0.07 ± 0.02 | 0.104 ± 0.011 | 0.05±0.01 | 0.25 ± 0.06 | 0.30 ± 0.13 |
| PS+N_4_P_2_K_2_ | 0.093 ± 0.010 | 0.06 ± 0.01 | 0.18 ± 0.09 | 0.05 ± 0.03 | 0.100 ± 0.005 | 0.06±0.02 | 0.32 ± 0.04 | 0.23 ± 0.07 |

Different letters indicate statistically significant differences between treatments at *p* < 0.05 (HSD test; one-way ANOVA).
